# Supplementary material for: The molecular transition that confers voltage dependence to muscle contraction
Source: Nat Commun. 2025 May 24;16:4847. doi: 10.1038/s41467-025-59649-7 (PMC12103506; doi:10.1038/s41467-025-59649-7)
Supplement: Supplementary file 1 — Supplementary Information [file 41467_2025_59649_MOESM1_ESM.pdf]

The Molecular Transition that Confers Voltage Dependence to Muscle Contraction

Marina Angelini, Nicoletta Savalli, Federica Steccanella, Savana Maxfield, Serena Pozzi,

Marino DiFranco, Stephen C. Cannon, Antonios Pantazis, Riccardo Olcese

SUPPLEMENTARY FIGURES &TABLES

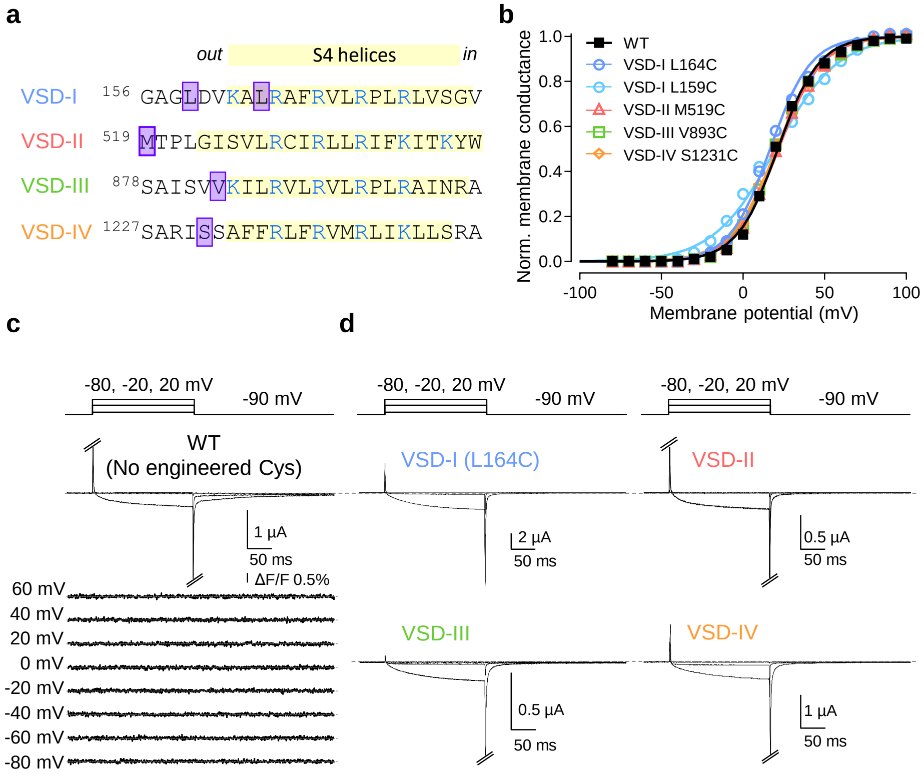

Supplementary Fig. 1: Sequence alignment of S4 segments and voltage dependence of activation for Ca$_V$1.1 WT and Cys mutants.

a, A sequence alignment of the S3-S4 loop and S4 segments in each VSD. Highlighted in blue are positively charged amino acids (Arg, R and Lys, K) within S4 helices (yellow background) that are compelled to move upon membrane depolarization driving VSD activation. Violet boxes indicate amino acids substituted into cysteines (Cys) for fluorescence labelling and voltage-clamp fluorometry. **b,** Mean voltage dependence of channel opening (G(V)) for WT and Cys mutants (L159C: n = 10, L164C: n = 6, M519C: n = 8, V893C: n = 14, S1231C: n = 7, WT: n = 7). Error bars (±SEM) are within the symbols when not visible. Note that the Cys mutations did not modify channel activation. Lines are fits to a Boltzmann distributions, fitting parameters are reported in Supplementary Table 1. **c,** Simultaneously recorded Ca^{2+} currents (top) and fluorescence signals (bottom) from WT channels without an engineered Cys. No voltage-dependent fluorescence changes were observed after labeling the channel with thiol-reactive fluorophores. **d,** Ionic currents from Cys mutants maintain WT-like properties.

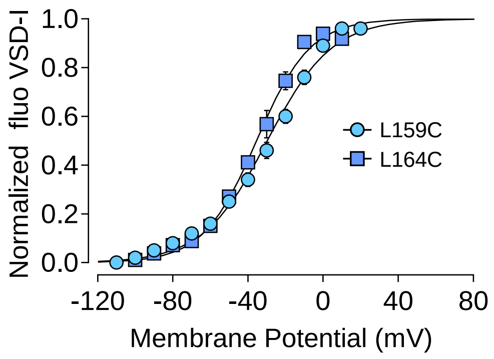

Supplementary Fig. 2: VSD-I Cys mutants used for VCF (L159C and L164C) share similar voltage-dependent properties.

Normalized F(V) curves for L159C and L164C obtained in 2mM Ba^{2+}. (L159C: $V_{1/2} = -30 \pm 2$ mV, n = 11, L164C: $V_{1/2} = -36 \pm 2$ mV, n = 6, L159C: $z = 1.6 \pm 0.04\ e_0$, L164C: $z = 1.9 \pm 0.1\ e_0$).

	$V_{1/2}$ (mV)	z (e_0)	n
**L159C**	19 ± 1	1.33 ± 0.02	10
**L164C**	17 ± 1	1.86 ± 0.05	6
**M519C**	22 ± 1	1.95 ± 0.04	8
**V893C**	20 ± 1	2.00 ± 0.04	14
**S1231C**	20 ± 1	1.86 ± 0.05	7
**WT**	20 ± 1	2.18 ± 0.06	7

Supplementary Table 1: Fitting parameters of the voltage-dependent activation of pore conductance.

Voltage dependence of pore conductance measured in 2mM Ba^{2+} was fitted to Boltzmann distribution (Supplementary Figure 1b). Values are reported as mean ± SEM. $V_{1/2}$ is half-activation potential, z is effective charge, and n is number of experiments.

	$V_{1/2}$ (mV)	z (e_0)	n
**VSD I**	−22 ± 1	2.2 ± 0.1	9
**VSD II**	8 ± 1	1.5 ± 0.1	5
**VSD III**	−24 ± 1	1.6 ± 0.04	10
**VSD IV**	−32 ± 2	0.6 ± 0.02	6
**G(V)**	29 ± 1	1.8 ± 0.1	6

Supplementary Table 2: Fitting parameters of the voltage-dependent activation of each VSD and pore.

Voltage dependence of VSD activation (fluorescence changes) and WT-channel pore conductance measured in 2mM Ca^{2+} were fitted to Boltzmann distributions (Fig. 3). Values are reported as mean ± SEM. $V_{1/2}$ is half-activation potential, z is effective charge, and n is number of experiments.

		Control	Nifedipine	*P*
**VSD I**	**$V_{1/2}$ (mV)**	−30 ± 2	−45 ± 2	<0.001
	**z (e_0)**	1.6 ± 0.04	1.2 ± 0.1	0.002
	**n**	11	5	
**VSD II**	**$V_{1/2}$ (mV)**	3 ± 1	1 ± 2	0.32
	**z (e_0)**	1.50 ± 0.06	1.35 ± 0.09	0.18
	**n**	8	5	
**VSD III**	**$V_{1/2}$ (mV)**	−25 ± 1	−43 ± 2	<0.001
	**z (e_0)**	1.71 ± 0.04	1.56 ± 0.04	0.012
	**n**	14	14	
**VSD IV**	**$V_{1/2}$ (mV)**	−32 ± 2	−27 ± 2	0.08
	**z (e_0)**	0.73 ± 0.03	0.71 ± 0.05	0.75
	**n**	7	7	

Supplementary Table 3: Fitting parameters of the voltage-dependent activation of each VSD in control or with nifedipine.

Voltage dependence of VSD activation (fluorescence changes) measured in 2mM Ba^{2+} were fitted to Boltzmann distributions (Fig. 4). Values are reported as mean ± SEM (two-tailed Student's *t*-tests). $V_{1/2}$ is half-activation potential, z is effective charge, and n is number of experiments, *P* is P-value.

Supplementary References

1 Wu, J. *et al.* Structure of the voltage-gated calcium channel Ca(v)1.1 at 3.6 A resolution. *Nature* **537**, 191-196, doi:10.1038/nature19321 (2016).
