# Supplementary material for: The molecular transition that confers voltage dependence to muscle contraction
Source: Nat Commun. 2025 May 24;16:4847. doi: 10.1038/s41467-025-59649-7 (PMC12103506; doi:10.1038/s41467-025-59649-7)
Supplement: Supplementary file 2 — Description of Additional Supplementary Files [file 41467_2025_59649_MOESM2_ESM.pdf]

Description of Additional Supplementary Files

File Name: Supplementary Movie 1

Description: Structural dynamics of CaV1.1 during activation by a skeletal-muscle action potential. Distinct functional domains of the human CaV1.1 channel structure (PDB ID:5GJV)1 were rendered so that their activities are shown as changes in brightness. The data were collected using VCF with action-potential clamp, using the skeletal-muscle AP waveform (Fig. 2b). Pore opening (conductance) is shown as white emission, while the activations of VSDs I, II, III and IV are shown in blue, red, green and orange, respectively.
